# Effect of Pemafibrate on Serum Creatinine in Patients with Chronic Kidney Disease

**DOI:** 10.31662/jmaj.2021-0212

**Published:** 2022-06-17

**Authors:** Enyu Imai, Atsuhiro Imai

**Affiliations:** 1 Nakayamadera Imai Clinic, Takarazuka, Japan; 2 Department of Nephrology, Fujita Medical University, Toyoake, Japan; 3 Department of Nephrology and Rheumatology, Aichi Medical University, Nagakute, Japan; 4 Department of Nephrology, Osaka University Graduate School of Medicine, Suita, Japan

**Keywords:** creatinine, renal function, fibrates, pemafibrate, CKD

## Abstract

**Introduction:**

Fibrates are recommended not to be used for the treatment of hypertriglyceridemia in patients with chronic kidney disease (CKD) based on clinical practice guidelines. The major reason for the negative suggestion is the elevation of serum creatinine and rhabdomyolysis by fibrates. This may cause clinical inertia for the treatment of hypertriglyceridemia using fibrate in patients with CKD, who are associated with an increasing risk of cardiovascular disease.

**Methods:**

We retrospectively studied the change of serum creatinine via the treatment of pemafibrate.

**Results:**

A total of 39 patients with CKD were treated with 0.2 mg of pemafibrate. Serum triglyceride was decreased in 23 fibrate-naïve patients from 380 [308, 455] mg/dL to 180 [152, 215] mg/dL via treatment with pemafibrate (p = 0.00003). Serum creatinine and eGFR were not changed from 1.22 ± 0.29 mg/dL to 1.21 ± 0.28 mg/dL (p = 0.70) and from 45.7 ± 10.9 mL/min/1.73 m^2^ to 46.2 ± 12.0 mL/min/1.73 m^2^ (p = 0.67) via treatment with pemafibrate, respectively. In 16 patients, with a change of treatment from fenofibrate or bezafibrate to pemafibrate, serum creatinine was significantly decreased from 1.32 ± 0.36 mg/dL to 1.17 ± 0.24 mg/dL (p = 0.003). eGFR was significantly increased from 45.2 ± 9.9 mL/min/1.73 m^2^ to 50.1 ± 8.6 mL/min/1.73 m^2^ (p = 0.001).

**Conclusions:**

These results suggest that treatment with pemafibrate does not affect the serum creatinine level and is suitable for use in patients with CKD for the treatment of hypertriglyceridemia.

## Introduction

Mild-to-moderately raised triglyceride (TG) concentration (2-10 mmol/L) at non-fasting conditions causes atherosclerosis but not at a greatly elevated concentration of TGs (>50 mmol/L) ^(1)^. Mild-to-moderately raised TG concentration is reported to be a risk factor for cardiovascular diseases ^(1), (2)^. Chronic kidney disease (CKD) is at a milieu of increasing TGs where apo CIII is increasing and lipoprotein is suppressed. Hypertriglyceridemia is associated with 15%-38% of patients with CKD ^(3), (4), (5)^. CKD is a strong risk factor for cardiovascular disease ^(6)^, and hypertriglyceridemia associated with CKD may be a cause of the high prevalence of cardiovascular diseases in this population.

Hypertriglyceridemia associated with CKD, however, is tended to clinical inertia because the administration of fibrates sometimes causes rhabdomyolysis ^(7), (8)^ and potentially deteriorates renal function, particularly, in patients with CKD ^(9), (10)^. Patients with CKD frequently have mixed dyslipidemia and require treatment with both statins and fibrates. Co-administration of fibrates with statins increases the risk of rhabdomyolysis ^(7)^, particularly in CKD ^(11)^. Guidelines for treatment of lipid abnormality in CKD do not recommend using fibrates for the treatment of hypertriglyceridemia ^(12), (13)^.

More than 20 mmol/L of TG concentration potentially causes acute pancreatitis ^(14)^ and is subjected to treatment with TGs lowering drugs. In real-world clinical practice, administration of fibrates to patients with CKD is limited to greatly elevated concentrations of TGs for prophylactic treatment of acute pancreatitis.

Increasing serum creatinine concentration has been reported by the administration of fenofibrate or bezafibrate ^(10), (15)^. By contrast, pemafibrate is reported to not affect the serum concentration of creatinine ^(16), (17)^. Nevertheless, these studies did not focus on renal function in patients with CKD.

We retrospectively studied the change of serum creatinine concentration after the administration of pemafibrate in patients with eGFR 20-60 mL/min/1.73 m^2^. Additionally, we investigate the change of serum concentration of creatinine via treatment with pemafibrate in patients treated with fenofibrate or bezafibrate.

## Materials and Methods

### Study design

We conducted a retrospective observational study. Nakayamadera Imai Clinic treated approximately 1600 patients a month, and 95% of them had chronic non-communicable diseases, mainly diabetes and CKD. In the clinic, patients whose triglyceridemia was higher than 300 mg/dL or higher than 200 mg/dL with metabolic syndrome were treated with fibrates, according to Third Report of the National Cholesterol Education Program Expert Panel on Detection, Evaluation, and Treatment of High Blood Cholesterol in Adults (NCEP-ATP III)^(18)^. One hundred and four patients with hypertriglyceridemia were treated with pemafibrate between June 2019 and May 2021. Among them, we enrolled 39 patients with CKD defined by GFR 20-60 mL/min/1.73 m^2^. We extracted clinical laboratory data from medical records. The clinical laboratory data were collected before and 2-3 months after treatment with pemafibrate, which included serum creatinine, TG, total cholesterol, HDL cholesterol, LDL cholesterol, AST, ALT, γ-GTP, blood pressure, and urinary protein concentration. These data were measured at the usual clinic visit in a non-fasting condition.

This study was approved by the ethics committee of Japan Medical Association (R3-03). Since this study is retrospective, we used the opt-out approach and the requirement for written informed consent was waived. This study was conducted according to the principles of the Declaration of Helsinki.

### Endpoints

The primary endpoint was the change of serum creatinine before and after the treatment of pemafibrate. We observed the change of serum creatinine separately in patients treated with or without fenofibrate or bezafibrate.

### Statistical analysis

The data are expressed in mean ± SD or median (interquartile range). The changes in clinical laboratory data were analyzed using paired t-test or Mann-Whitney U test; p < 0.05 indicates statistical significance. All analyses were performed using R 4.0.3.

## Results

A total of 39 patients with CKD were treated with 0.2 mg of pemafibrate. Table 1 shows the clinical characteristics of the patients at baseline. Among them, 16 patients treated with pemafibrate were switched from 80 to 100 mg of fenofibrate (n = 13) or from 200 to 400 mg of bezafibrate (n = 3) and 23 patients were naïve for fibrate. Seventeen patients were treated with statins.

**Table 1.** Baseline Characteristics of CKD Patients Treated with Pemafibrate (n = 39).

	All	Fibrate naïve	Treated with fibrates	p values
	(n = 39)	(n = 23)	(n = 16)	
Age (y.o.)	65 ± 10	67 ± 9	63 ± 12	0.19
Male (n(%))	31 (79.5)	17 (73.9)	14 (87.5)	0.432
CKD Stage 3a (n(%))	19 (48.7)	11 (47.8)	8 (50.0)	1.0
CKD Stage 3b (n(%))	17 (43.6)	10 (43.4)	7 (43.8)	1.0
CKD stage 4 (n(%))	3 (7.7)	2 (8.7)	1 (6.3)	1.0
Diabetes Mellitus (n(%))	14 (36.9)	6 (26.1)	8 (50.0)	0.23
Hypertension (n(%))	35 (89.7)	20 (87.0)	15 (93,7)	0.88
CVD (n(%))	5 (12.8)	3 (13.0)	2 (12.5)	1.0
Body weight (kg)	74.7 ± 13.2	74.3 ± 11.7	75.2 ± 15.5	0.84
BMI (kg/m^2^)	27.0 ± 4.0	27.2 ± 4.0	26.6 ± 4.2	0.67
Systolic blood pressure (mmHg)	131 ± 14.8	131 ± 14.5	132 ± 15.7	0.89
Serum creatinine (mg/dL)	1.26 ± 0.32	1.22 ± 0.29	1.32 ± 0.36	0.36
eGFR (mL/min/1.73 m^2^)	45.5 + 10.4	45.7 + 10.9	45.2 + 9.9	0.87
Triglyceride (mg/dL)	322 [206, 438]	380 [308, 455]	202 [170, 304]	0.001
Total cholesterol (mg/dL)	200 + 37.6	206 + 42.1	191 + 29.6	0.24
LDL-cholesterol (mg/dL)	107.4 + 26.7	106.1 + 28.9	109.2 + 24.1	0.73
HDL-cholesterol (mg/dL)	45.0 + 9.3	45.0 + 8.0	45.0 + 11.2	1.0
Treatment with bezafibrate (n(%))			3 (18.8)	
Treatment with fenofibrate (n(%))			13 (81.2)	
Treatment with statins (n(%))	17 (43.6)	10 (43.4)	7 (43.8)	

LDL cholesterol: low-density lipoprotein cholesterol; HDL cholesterol: high-density lipoprotein cholesterol

**Figure 1.**
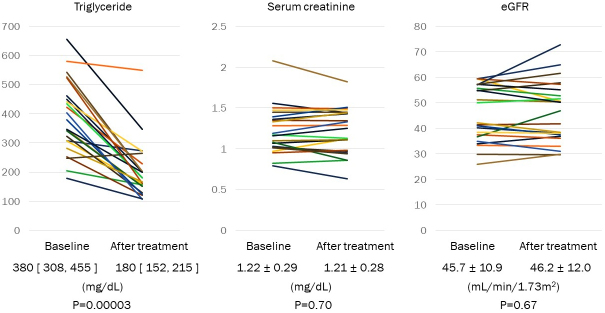
Change of triglyceride, serum creatinine, and eGFR in fibrate-naïve patients with CKD. Fibrates-naïve patients with hypertriglyceridemia were treated with pemafibrate. Baseline and 2-3 months after treatment with pemafibrate, blood sampling was performed in non-fasting condition at clinic visit. Data are shown in mean ± SD or the median [25%, 75%].

TG was decreased in fibrate-naïve patients from 380 [308, 455] mg/dL to 180 [152, 215] mg/dL by treatment with pemafibrate (p = 0.00003) (Figure 1). HDL cholesterol was significantly increased from 45.0 ± 8.0 mg/dL to 56.1 ± 9.8 mg/dL (p < 0.0001). Serum creatinine and eGFR were not changed from 1.22 ± 0.29 mg/dL to 1.21 ± 0.28 mg/dL (p = 0.70) and from 45.7 ± 10.9 mL/min/1.73 m^2^ to 46.2 ± 12.0 +11.8 mL/min/1.73 m^2^ (p = 0.67) via treatment with pemafibrate, respectively (Figure 1).

**Figure 2.**
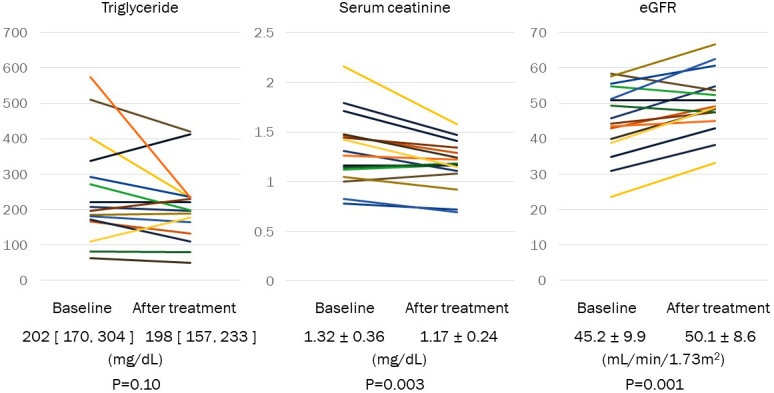
Change of triglyceride, serum creatinine, and eGFR in patients with CKD treated with 200-400 mg of bezafibrate or 80-100 mg of fenofibrate. Fibrate-treated patients with hypertriglyceridemia were shifted to pemafibrate. Baseline and 2-3 months after treatment with pemafibrate, blood sampling was performed in non-fasting condition at clinic visit. Data are shown in mean ± SD or the median [25%, 75%].

In patients, with a change of treatment from fenofibrate or bezafibrate to pemafibrate, TG and HDL cholesterol were not changed from 202 [170, 304] mg/dL to 198 [157, 233] mg/dL (p = 0.10) and from 45 + 11mg/dL to 48 + 12 mg/dL (p = 0.079), respectively (Figure 2 and Table 2). Serum creatinine was significantly decreased from 1.32 ± 0.36 mg/dL to 1.17 ± 0.24 mg/dL (p = 0.003). eGFR was significantly increased from 45.2 ± 9.9 mL/min/1.73 m^2^ to 50.1 ± 8.6 mL/min/1.73 m^2^ (p = 0.001) (Figure 2).

**Table 2.** Effect of Pemafibrate Treatment on Liver Enzyme and Lipid.

	Baseline	After treatment	p values	
AST (U/L)	23.5 [20.0, 33.5]	26.5 [21.0, 31.0]	0.94
ALT (U/L)	24.5 [18.3, 47.0]	25.5 [15.5, 36.3]	0.006
g-GTP (U/L)	43.0 [25.0, 73.5]	25.5 [22.0, 53.3]	0.00001
Total cholesterol (mg/dL)	199.6 ± 37.6	192.1 ± 25.9	0.17	
LDL-cholesterol (mg/dL)	107.4 ± 26.7	108.4 ± 20.0	0.82	
HDL-cholesterol (mg/dL)	45.0 ± 9.3	52.7 ± 11.3	<0.00001
Triglyceride (mg/dL)	322 [206, 438]	188 [151, 232]	<0.00001

AST: aspartate aminotransferase; ALT: alanine aminotransferase; γ-GTP: γ-glutamyl transferase; LDL cholesterol: low-density lipoprotein cholesterol; HDL-cholesterol: high-density lipoprotein cholesterol

**Figure 3.**
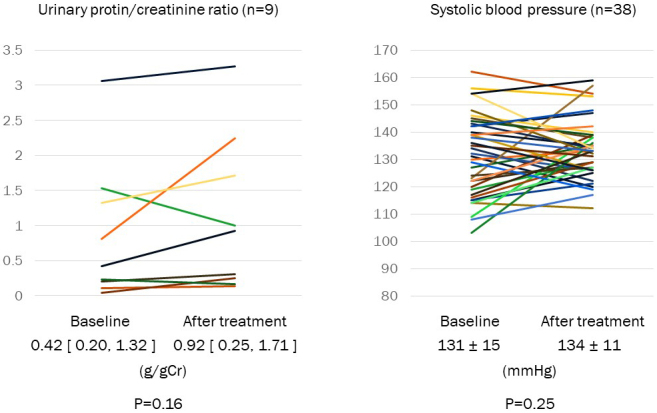
Change of urinary protein/creatinine ratio and systolic blood pressure. Urinary protein/creatinine ratio was measured at baseline and after treatment with pemafibrate in patients with proteinuria (n = 9). Systolic blood pressure was shown at baseline and after treatment (n = 38). Data were shown in mean ± SD or the median [25%, 75%].

ALT and γ-GTP were significantly decreased after treatment with pemafibrate (Table 2). AST, blood pressure, and urinary protein/creatinine ratio were not changed via the treatment of pemafibrate (Figure 3). No patient had rhabdomyolysis during the study period.

## Discussion

In the present study, serum creatinine concentration was not changed by the administration of pemafibrate in patients with eGFR 20-60 mL/min/1.73 m^2^ as consistent with previous studies including patients with normal renal function and CKD. In patients treated with fenofibrate or bezafibrate, serum creatinine concentration was decreased by 0.15 mg/dL after shifting to pemafibrate. Similarly, eGFR was increased by approximately 10% from mean eGFR 45 mL/min/1.73 m^2^.

In a Japanese evidence-based clinical practice guideline for CKD 2018 ^(13)^ as well as in KDIGO clinical practice guideline for lipid management 2013 ^(12)^, treatment of hypertriglyceridemia with fibrates is not recommended. As a basis, these guidelines cited a large observational study that found that prescriptions for fibrate significantly increased sCr levels, as well as the risk of hospitalization and nephrologist consultation ^(10)^. These guidelines only suggest the change of lifestyle such as dietary modification, weight reduction, increased physical activity, reducing alcohol intake, and treatment of hyperglycemia (if present). Additionally, fibrates may cause rhabdomyolysis, in particular, in the CKD population. Not only general practitioners but also nephrologists hesitate to use fibrates, although they are aware of the potential benefit of lipid-lowering therapy with fibrates for preventing the incidence and recurrence of CVD events. In meta-analyses, fibrates significantly reduced the risk of cardiovascular death, non-fatal myocardial infarction, and non-fatal stroke, in primary and secondary prevention ^(19), (20)^. We think that there is a clinical inertia in the treatment of hypertriglyceridemia in CKD. Pemafibrate, which does not affect the serum creatinine concentration in patients with CKD, may break through the inertia. A lower incident rate of drug reaction of pemafibrate with statin than that of fenofibrate ^(16)^ could encourage physicians to treat hypertriglyceridemia in CKD.

The present study is a retrospective observational study. We cannot elucidate the cause of increasing serum creatinine via treatment with fenofibrate or bezafibrate but not via treatment with pemafibrate. Nevertheless, in the literature of previous studies, it is apparent that the glomerular filtration rate was not decreased by fibrates. Ansquer and colleagues ^(15)^ carefully demonstrated that fibrates did not reduce the inulin clearance in patients with normal renal function. By contrast, creatinine clearance was decreased by 10% ^(15)^, suggesting increasing creatinine synthesis and/or reduction of excretion of creatinine from the proximal tubule. Cystatin C, which is degraded in the proximal tubule, is also increased by treatment with fenofibrate ^(16)^. This may support that fenofibrate attenuates proximal tubule function transiently since serum creatinine and cystatin C returned to the baseline level after discontinuance. Increasing urinary creatinine excretion was observed in the study ^(15)^, which may also cause an increasing level of serum creatinine.

Non-fasting hypertriglyceridemia is associated with myocardial infarction, stroke, and all-cause death ^(1)^. CKD is a strong risk factor for cardiovascular diseases ^(6)^. SHARP study ^(21)^ showed that reduction of LDL cholesterol resulted in a 17% reduction of cardiovascular diseases. Nevertheless, 10% of the participants of the SHARP study developed CVD in 5 years of follow-up. Hence, reduction of LDL cholesterol alone is not sufficient for secondary prevention of CVD. This may suggest that the remaining hypertriglyceridemia may play an important role in the high incidence of CVD after treatment for LDL cholesterol. In a meta-analysis, lowering of hypertriglyceridemia showed a reduction of CVD in patients with CKD ^(10)^.

In a meta-analysis, the administration of fibrates was reported to reduce proteinuria ^(10)^. Treatment with pemafibrate also showed a reduction of albuminuria in IgA patients ^(22)^. In the present study, a reduction of proteinuria was not observed. However, it is hard to conclude since our study is a short duration of treatment and the number of patients was small. Further study is required.

Reduction of ALT and γ-GTP levels by pemafibrate was previously reported in patients with CKD ^(17)^, as well as in those without CKD ^(16)^.

The limitations of our study are as follows. This is a retrospective observational study conducted in a single center. We draw data from medical records during clinical practice. Cystatin C is not available for a marker of renal function in general practice because we cannot measure it in less than 3 months under practice according to national insurance.

### Conclusion

Serum creatinine concentration was not changed via the administration of pemafibrate in patients with eGFR 20-60 mL/min/1.73 m^2^. In patients treated with fenofibrate or bezafibrate, serum creatinine concentration was decreased by 0.15 mg/dL after the switch to pemafibrate. Similarly, eGFR was increased by approximately 10% from mean eGFR 45 mL/min/1.73 m^2^. Pemafibrate significantly decreased serum TGs equivalent to fenofibrate or bezafibrate in patients with CKD. These results suggest that pemafibrate is suitable for use in patients with CKD for the treatment of hypertriglyceridemia. To investigate the impact of pemafibrate on primary and secondary prevention of cardiovascular diseases in patients with CKD, further study is warranted.

## Article Information

### Conflicts of Interest

Enyu Imai received honorarium from Boehringer Ingelheim, Daiichi Sankyo, Lilly, Novo, and Bayer; Enyu Imai received research fundings from Tanabe Mitsubishi, Boehringer Ingelheim, Kissei, Kyowa Kirin, Daiichi Sankyo, and AstraZeneca.

### Author Contributions

Enyu Imai has responsibility regarding all of the study.

Atsuhiro Imai conducted statistics of this study.

### Approval by Institutional Review Board (IRB)

This study was approved by the ethics committee of Japan Medical Association (R3-03).
